# Appointmentments in Medical Schools

**Published:** 1859-03

**Authors:** 


APPOINTMENTS IN MEDICAL SCHOOLS.—Dr. A. G. Thomas has been appointed Professor of Physiology in the Oglethorpe Medical College, Georgia.

Dr. John C. Draper has been appointed to the Professorship of Analytical and Practical Chemistry in the University of New York.

